# Exercise training attenuates pulmonary inflammation and mitochondrial dysfunction in a mouse model of high-fat high-carbohydrate-induced NAFLD

**DOI:** 10.1186/s12916-022-02629-1

**Published:** 2022-11-08

**Authors:** Jinkyung Cho, Bruce D. Johnson, Kymberly D. Watt, Alexander S. Niven, Dongwook Yeo, Chul-Ho Kim

**Affiliations:** 1 Department of Cardiovascular Disease, Mayo Clinic, 200 First St. SW, Rochester, MN 55905 USA; 2 Department of Sport Science, Korea Institute of Sport Science, Seoul, Republic of Korea; 3 Department of Gastroenterology and Hepatology, Mayo Clinic, Rochester, MN USA; 4 Department of Pulmonary and Critical Care Medicine, Mayo Clinic, Rochester, MN USA

**Keywords:** Exercise, NAFLD, Pulmonary inflammation, Mitochondrial function, Mitochondrial dynamics

## Abstract

**Background:**

Non-alcoholic fatty liver disease (NAFLD) can lead to pulmonary dysfunction that is associated with pulmonary inflammation. Moreover, little is known regarding the therapeutic role of exercise training on pulmonary pathophysiology in NAFLD. The present study aimed to investigate the effect of exercise training on high-fat high-carbohydrate (HFHC)-induced pulmonary dysfunction in C57BL/6 mice.

**Methods:**

Male C57BL/6 mice (*N* = 40) were fed a standard Chow (*n* = 20) or an HFHC (*n* = 20) diet for 15 weeks. After 8 weeks of dietary treatment, they were further assigned to 4 subgroups for the remaining 7 weeks: Chow (*n* = 10), Chow plus exercise (Chow+EX, *n* = 10), HFHC (*n* = 10), or HFHC plus exercise (HFHC+EX, *n* = 10). Both Chow+EX and HFHC+EX mice were subjected to treadmill running.

**Results:**

Chronic exposure to the HFHC diet resulted in obesity with hepatic steatosis, impaired glucose tolerance, and elevated liver enzymes. The HFHC significantly increased fibrotic area (*p* < 0.001), increased the mRNA expression of TNF-α (4.1-fold, *p* < 0.001), IL-1β (5.0-fold, *p* < 0.001), col1a1 (8.1-fold, *p* < 0.001), and Timp1 (6.0-fold, *p* < 0.001) in the lung tissue. In addition, the HFHC significantly altered mitochondrial function (*p* < 0.05) along with decreased Mfn1 protein levels (1.8-fold, *p* < 0.01) and increased Fis1 protein levels (1.9-fold, *p* < 0.001). However, aerobic exercise training significantly attenuated these pathophysiologies in the lungs in terms of ameliorating inflammatory and fibrogenic effects by enhancing mitochondrial function in lung tissue (*p* < 0.001).

**Conclusions:**

The current findings suggest that exercise training has a beneficial effect against pulmonary abnormalities in HFHC-induced NAFLD through improved mitochondrial function.

**Supplementary Information:**

The online version contains supplementary material available at 10.1186/s12916-022-02629-1.

## Background

Non-alcoholic fatty liver disease (NAFLD) refers to abnormal fat accumulation in the liver that is not associated with excessive alcohol intake. The prevalence of NAFLD is gradually increasing, with approximately 75~100 million individuals affected by this disease in the USA in 2015 [1]. Although it begins initially as asymptomatic fatty liver, NAFLD can progress to more severe diseases including non-alcoholic steatohepatitis (NASH), cirrhosis, and hepatocellular carcinoma with a mortality rate of 30~40% [2]. Furthermore, NAFLD appears to have several pathogenic connections to lung disease [3]. A number of epidemiologic studies have reported that NAFLD is associated with impairments in lung function. These previous studies reported that forced vital capacity (FVC) and forced expiratory volume in 1 sec (FEV_1_) were significantly reduced in hepatic steatosis relative to non-steatotic control, and moreover, FVC and FEV_1_ were inversely related to the severity of hepatic steatosis [4, 5]. In addition, NAFLD was associated with chronic obstructive pulmonary disease [6, 7], obstructive sleep apnea [6], and, in more severe cases, hepatopulmonary syndrome and portopulmonary hypertension [8]. The postulated pathogenic underlying mechanisms are complicated; however, preliminary studies suggest that excessive fat accumulation may lead to hepatic inflammation and mitochondrial dysfunction due to the upregulation of inflammatory cytokines and oxidative stress [9–11]. Since the liver is linked in series with the portal and the pulmonary systems, chronic increases in inflammatory cytokines, hepatic oxidative stress, and toxic metabolites that are bypassing the liver may directly and/or indirectly influence the pulmonary system, facilitating a systemic inflammatory response in the lungs [12–14].

The mitochondria have a pivotal role, not only in energy production but also in innate immune responses. Therefore, mitochondrial homeostasis is vital for cell fitness and cell fate. In addition, mitochondrial function is also a significant modulator in the inflammatory process. It is commonly characterized by a decrease in oxidative phosphorylation system (OXPHOS) and an increase in reactive oxygen species (ROS) production, which eventually can provoke inflammatory progression [15]. In NAFLD, mitochondrial dysfunction is involved in the hepatic inflammatory process and appears to be significantly associated with NAFLD disease progression [16, 17]. In addition, a decline in mitochondrial function is linked to diverse types of pulmonary disease including cystic fibrosis, chronic obstructive pulmonary disease (COPD), and pulmonary arterial hypertension, which can be observed in NAFLD [18]. Nevertheless, there is little known about pulmonary inflammation and mitochondrial function in NAFLD.

NAFLD is generally caused by insulin resistance and metabolic syndrome related to an unbalanced diet, physical inactivity, obesity, and hypertension [19, 20]. Accordingly, exercise is often recommended as a non-pharmacological strategy for alleviating the risk factors of metabolic syndrome [21, 22]. Exercise can not only downregulate inflammatory cytokines but also promotes mitochondrial biogenesis and enhances morphological dynamics and turnover rate via biogenesis and mitophagy [23, 24]. Recently, we found that exercise was able to reduce insulin resistance [25] and reduce hepatic fat accumulation and inflammation levels in NAFLD [26]. To extend these findings, the present study evaluated inflammation, histological formation, and mitochondrial function of pulmonary tissue in a high-fat high-carbohydrate (HFHC)-induced animal model of NAFLD and examined if 7 weeks of exercise training could improve those variables.

## Methods

### Animals

**Fig. 1:**
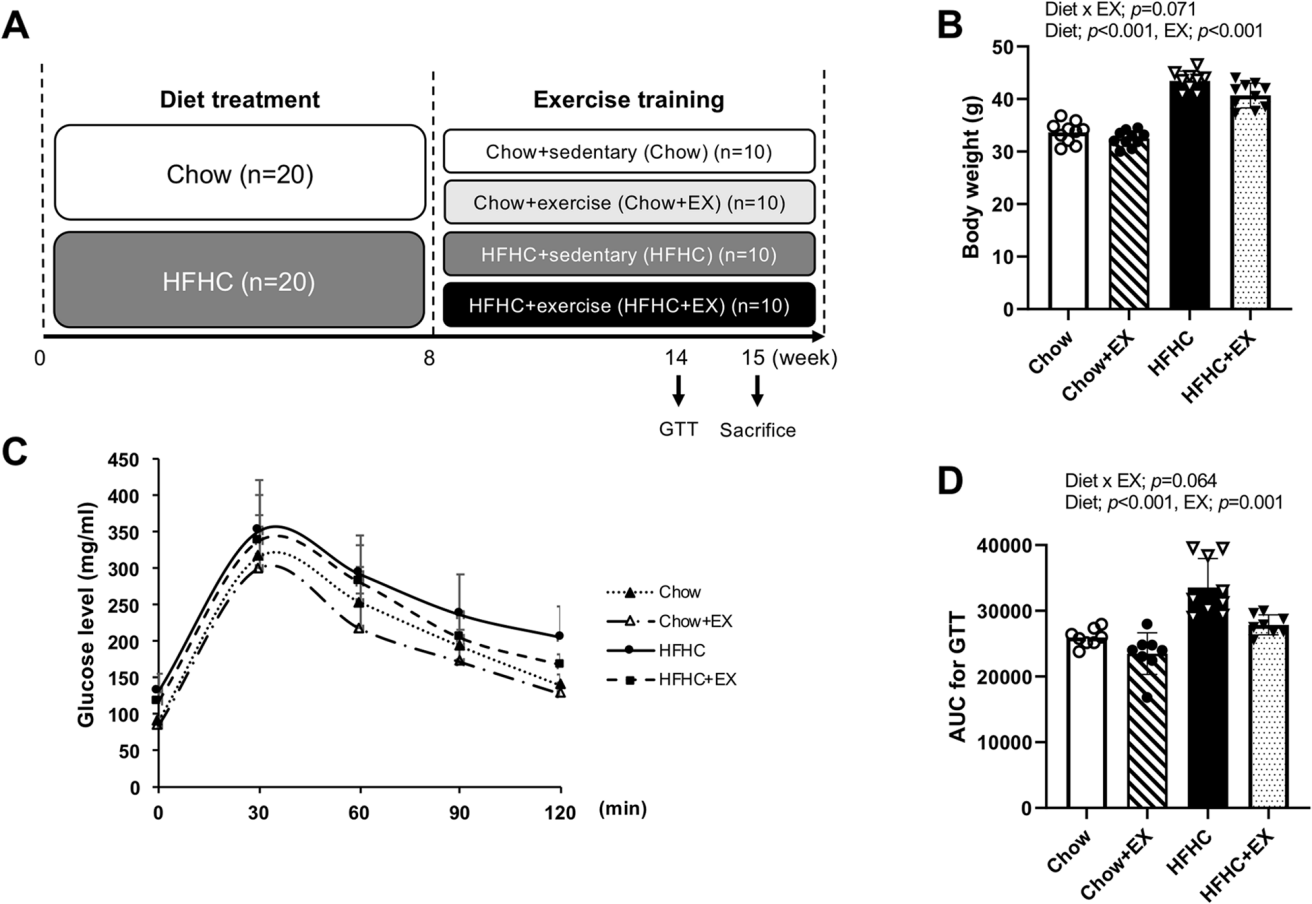
Treadmill running improved the metabolic status in HFHC-induced C57BL/6 mice. **A** Study design. **B** Body weight at the end of the experimental period (*n* = 8–10 per group). **C**, **D** Glucose tolerance test (GTT) and corresponding area under the curve (AUC) values (*n* = 8–10 per group). Data are presented as mean ± S.D. Statistical analyses were performed by two-way ANOVA. Post hoc *t*-tests were performed to compare the differences between the 2 groups, if needed

Forty male C57BL/6 mice aged 8 weeks were purchased from ORIENT BIO Inc. (Seongnam, Republic of Korea). Mice were randomly divided into the standard diet group (Chow, *n* = 20) or the high-fat high-carbohydrate diet group (HFHC, *n* = 20) (Fig. 1A). The Chow diet group was fed a control diet (Purina Mills, Seoul, Republic of Korea) consisting of 10% fat (by kcal), 70% carbohydrates (by kcal), and 20% protein (by kcal). The HFHC diet group was fed a high-fat high-carbohydrate diet (D12331, Research Diet, NJ, USA) consisting of 58% fat (by kcal), 25% carbohydrates (by kcal), and 17% protein (by kcal) with drinking water mixed with 42 g/L of carbohydrates at a ratio of high fructose (Junsei, Japan, CAS 57-48-7) and sucrose (Sigma-Aldrich, St. Louis, MO, S1888, CAS 57-50-1).

Mice were housed under controlled light (12:12-h light-dark cycle starting at 8:00 A.M.), humidity (50 ± 5%), and temperature (20 ± 1 °C) with free access to food and water ad libitum. This study was approved by the Sungkyunkwan University Institutional Animal Care and Use Committee (IACUC). All animal procedures and care were conducted with approved animal protocols in accordance with the IACUC guidelines. Efforts were made to minimize the pain or distress experienced by laboratory animals.

### Experimental design

Figure 1A illustrates the study’s experimental design. Male C57BL/6 mice were randomly divided into the Chow diet group (*n* = 20) or the HFHC diet group (*n* = 20) for 15 weeks. After 8 weeks of dietary treatment, both the Chow diet group and the HFHC diet group were further assigned to four groups for the remaining 7 week period: Chow (*n* = 10), Chow plus exercise (Chow+EX, *n* = 10), HFHC (*n* = 10), and HFHC plus exercise (HFHC+EX, *n* = 10). The exercise groups ran on a motor-driven rodent treadmill (Columbus Instruments, Columbus, OH, USA) 5 times/week for 7 weeks. After 1week of the exercise adaptation period, mice exercised on a treadmill for 50 min at a fixed speed of 14 m/min with 0° inclination. This exercise intensity is the moderate level which is approximately 60–80% of maximal oxygen consumption (VO_2max_) based on a percentage of VO_2max_ index [27, 28]. During exercise sessions, if necessary, mouse tails were gently tapped to encourage exercise, and no electrical shock was administered. To avoid the acute effect of exercise training, sacrifice was performed 48 h after the last exercise training. Body weight was monitored weekly. Mice were not excluded during the procedure.

### Glucose tolerance test (GTT)

Mice were fasted for 16 h and underwent a glucose tolerance test (GTT) consisting of the administration of 1.5 g glucose/kg body weight via intraperitoneal (IP) injection (Sigma-Aldrich, MO, USA). The blood glucose level was measured at 0 (fasting glucose), 15, 30, 45, 60, and 120 min after glucose IP injection from the tail vein blood using a glucometer (One Touch, Life Scan, NJ, USA). The area under the curve (AUCs) of the GTT was determined using a linear trapezoidal method.

### Blood and tissue sampling

At the end of treatment (diet and exercise), blood and tissues were collected from mice after overnight fasting. Blood was collected from the hepatic portal vein right before sacrificing the mice under anesthesia with diethyl ether. Blood was centrifuged at 3000 rpm and 4 °C for 10 min and stored at – 80 °C in a freezer until analysis. The liver and the right upper lobe of lung tissues were rapidly excised and flash-frozen in liquid nitrogen. For mitochondrial O_2_ respiration and H_2_O_2_ emission, analysis was performed with fresh lung tissue immediately post-sacrifice.

### Biochemical analyses

Serum concentrations of hepatic enzymes including aspartate transaminase (AST) and alanine aminotransferase (ALT) were determined by standard procedures with a Beckman DXC800 analyzer (Beckman, Brea, CA, USA).

### Histology

Liver and right lung parenchyma tissues were fixed in 10% buffered formalin (Sigma Aldrich, MO, USA) for 24 h and then embedded in paraffin. Embedded tissues were sectioned in the midline at 5-μm-thick slices on a microtome (CM3050S, Leica Microsystems, Nussloch, Germany) and stained with hematoxylin and eosin (H&E) and Masson’s trichrome for histological evaluation. A blinded expert was provided with the stained sections and took images of them with a Leica DMLS light microscope (Leica Microsystems). The percentage of fibrotic lung tissues was determined using the ImageJ software (National Institutes of Health, Bethesda, MD, USA). The data were shown as mean ± S.D.

### NAFLD activity score (NAS)

The severity of NAFLD was assessed using the NAS score, which was validated by the Nonalcoholic Steatohepatitis Clinical Research Network [29]. The NAS score was calculated as the sum of steatosis (< 5% = 0; 5–33% = 1; 33–66% = 2; > 66% = 3), lobular inflammation (none = 0; < 2 foci = 1; 2–4 foci = 2; > 4 foci = 3), and ballooning (none = 0; few = 1; prominent = 2). A NAS < 3 is considered simple steatosis, and a NAS > 5 is considered NASH.

### Liver lipid isolation and quantification

Hepatic lipids were isolated using the method of Folch et al. [30]. The level of liver triglyceride (TG) was measured using enzymatic assays in commercial kits from Wako Chemicals (Richmond, VA, USA).

### Real-time PCR

Total RNA was extracted from tissues using RNA extraction kits (Applied Biosystems, Foster City, CA, USA) according to the manufacturer’s protocol. The quantity and quality of total RNA were determined using the absorbance ratios at 260/230 and 260/280 through Nanodrop 2000 spectrophotometer (Thermo Scientific, Brookfield, WI, USA). Fifty nanograms of total RNA was mixed with a master mixture using TaqMan® RNA-to-Ct™ 1-Step Kit and performed in an ABI prism 7500 real-time System (Applied Biosystems). Commercial FAM-labeled TaqMan probes (Applied Biosystems) were used to measure tumor necrosis factor-α (TNF-α, Mm00443258_m1), interleukin-1β (IL-1β, Mm Mm00434228_m1), TIMP metallopeptidase inhibitor 1 (Timp1, Mm01341361_m1), collagen type I alpha 1 chain (Col1a1, Mm00801666_g1), and beta-actin (β-actin, Mm02619580_g1). A β-actin was used as a reference gene for the lungs [31, 32]. Target gene expression was normalized against the expression of β-actin. Expression levels were calculated using the comparative cycle time method. Each experiment was performed in triplicate.

### Mitochondrial O_2_ respiration

Lung tissues were homogenized with assay buffer (buffer Z with 50 μmol/L EGTA + 20 mmol/L creatine) and transferred to a polarographic high-resolution respirometer chamber using an Oxygraph-2k; O2k (OROBOROS, Innsbruck, Austria). Electron flow through complex I (5 mmol/L glutamate + 2 mmol/L malate) was measured to check the leak oxygen consumption. Following stabilization, 4 mmol/L adenosine diphosphate (ADP) was added to measure the oxidative capacity, and 10 mmol/L succinate was subsequently added for complex II electron flow. The level of mitochondrial O_2_ respiration was normalized against lung tissue weight.

### Mitochondrial H_2_O_2_ emission

The assay based on Amplex Red was used to measure the emission of hydrogen peroxide (H_2_O_2_) from lung tissues. The assay was measured using a Spex FluoroMax 4 spectrofluorometer (Horiba Scientific, Edison, NJ) (excitation at 563nm, emission at 585nm) maintained at 37 °C during state 4 respiration (10 μg/mL oligomycin). The oxidation of Amplex Red was continuously monitored using the following protocol: 10 μM Amplex Red, 1 U/mL horseradish peroxidase, and 10 μg/mL oligomycin settings and 1 mM malate + 2 mM glutamate (complex I substrates), 3 mM succinate (complex II substrate), and 10 mM glycerol-3-phosphate (lipid substrate). Before the assay, a standard curve for the relationship between Amplex Red fluorescence and H_2_O_2_ was determined. The H_2_O_2_ emission rate after removing the background value from each of the standard values was calculated from the Δ*F*/min gradient values and then expressed as pmol min^−1^ mg^−1^ tissue weight.

### Western blot

Lung tissues were homogenized with lysis buffer consisting of 50 mM Tris–HCl (pH 7.5), 150 mM NaCl, 0.5% deoxycholic acid, 1% Nonidet P40, 0.1% sodium dodecyl sulfate, 1 mM PMSF, and leupeptin 100 mg/mL. Homogenates were centrifuged, and the supernatant was taken. The protein concentrations were checked by the Bradford assay (Bio-Rad, Hercules, CA, USA). Ten to 15 μg of total proteins was loaded on 7.5–15% sodium dodecyl sulfate/polyacrylamide gel electrophoresis, transferred to nitrocellulose membranes, and blocked in 5% skim milk in Tris-buffered saline with 0.1% Tween-20. The membranes were incubated overnight at 4 °C with primary antibodies for β-actin (1:1000 dilution; Bethyl Laboratories, Montgomery, TX, USA), Mitofusin 1(1:300 dilution; Mfn1; sc-166644; Santa Cruz Biotechnology, Paso Robles, CA, USA), Mitofusin2 (1:300 dilution; Mfn2; #9482; Cell Signaling, Danvers, MA, USA), or Fis1 (1:300 dilution; sc-376447; Santa Cruz Biotechnology) and subsequently incubated with horseradish peroxidase (HRP)-conjugated secondary antibodies at room temperature for 1 h. Finally, blots were visualized with a chemiluminescent HRP substrate kit (Millipore, Billerica, MA, USA) via ChemiDoc (Bio-Rad). A β-actin (Bethyl Laboratories) antibody was used as an internal control. The intensity of the bands was determined by the ImageJ software (National Institutes of Health).

### Statistical analysis

All values are expressed as the mean ± standard deviation. Two-way ANOVA was performed to determine the main effect of diet (Chow vs. HFHC diet), the main effect of exercise training (sedentary vs. exercise), and the interaction between diet and exercise training. Subsequently, post hoc *t*-tests were performed to compare the differences between the 2 groups, if needed. Statistical analyses were performed using the SPSS software (version 24.0 for Windows, SPSS, Inc., Chicago, IL, USA). *P* values < 0.05 were considered statistically significant. The present study is reported in accordance with the ARRIVE guidelines (Supplementation).

## Results

### Weight gains and glucose tolerance

Figure 1B shows the mean body weight for the 4 experimental groups at the end of the experimental period. The HFHC diet groups (HFHC, HFHC+Ex) showed significantly higher body weight relative to the Chow diet groups (Chow, Chow+Ex) (*p* < 0.001). Additionally, the exercise groups (Chow+Ex, HFHC+Ex) demonstrated significantly lower body weight relative to the sedentary groups (Chow, HFHC) (*p* < 0.001). There was a trend of significant differences in body weight among groups (*p* = 0.071). For insulin resistance, the HFHC diet groups (HFHC, HFHC+Ex) showed significantly impaired glucose tolerance relative to the Chow diet groups (Chow, Chow+Ex) (*p* < 0.001). Moreover, the exercise groups (Chow+Ex, HFHC+Ex) demonstrated greater glucose tolerance than the sedentary groups (Chow, HCHC) (*p =* 0.001). There was a trend of significant differences in glucose tolerance among groups (Fig. 1C, D, *p* = 0.064).

### Hepatic steatosis and liver damage markers in HFHC-induced NAFLD

**Fig. 2:**
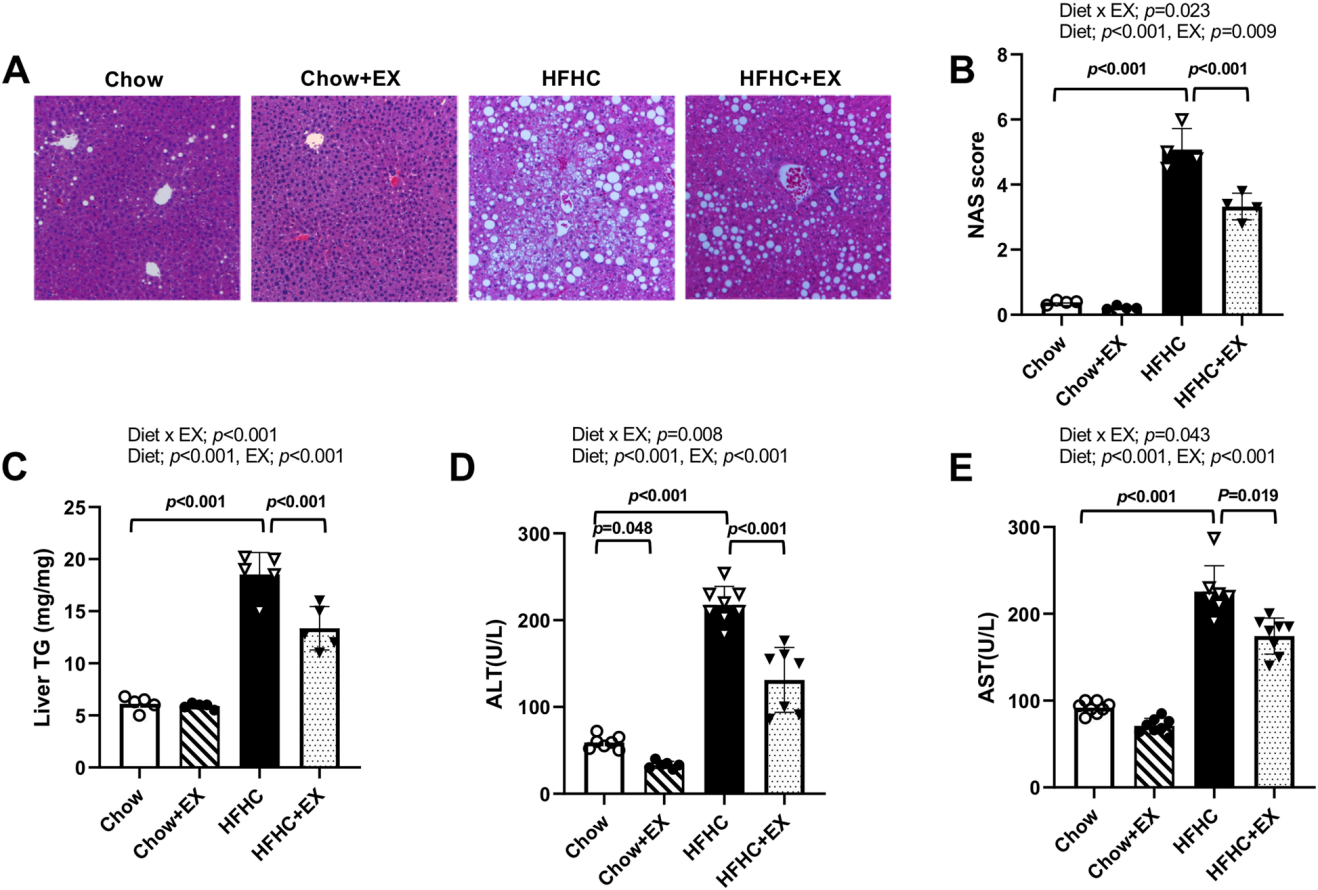
Treadmill running attenuated the hepatic steatosis and its damage markers in HFHC-induced mice. **A** Representative liver H&E staining (*n* = 4 per group). Scale bar represents 100 μm. **B** NAS score (*n* = 4 per group). **C** Liver TG (*n* = 5 per group). **D** Serum ALT and **E** AST levels (*n* = 8–10 per group). Data are presented as mean ± S.D. Statistical analyses were performed by two-way ANOVA. Post hoc *t*-tests were performed to compare the differences between the 2 groups, if needed

To examine whether prolonged HFHC induced NAFLD, H&E staining of the liver tissue was performed. The severity of the NAFLD was evaluated by NAS (Fig. 2A, B). There were significant differences in NAFLD severity among the groups (*p =* 0.023). The HFHC displayed extensive steatosis and lobular inflammation with ballooning (mean 5.00 ± 0.29 vs. 0.28 ± 0.48 of HFHC vs. Chow, respectively, *p* < 0.001). For fat accumulation in the liver, there were significant differences in TG contents among the groups (*p* < 0.001). The HFHC showed significantly greater amounts of intrahepatic TG contents (mean 18.38 ± 1.73 vs. 5.85 ± 0.74 mg/mg of HFHC vs. Chow, respectively, *p* < 0.001) than the Chow (Fig. 2C). With respect to liver damage markers, there were significant differences in ALT and AST among the groups (*p* < 0.001). The HFHC showed significantly higher levels of serum ALT (mean 218.3 ± 24.9 vs. 59.0 ± 8.4 U/L of HFHC vs. Chow, respectively, *p* < 0.001) and AST (mean 229.8 ± 35.2 vs. 91.2 ± 7.4 U/L of HFHC vs. Chow, respectively, *p* < 0.001) than the Chow (Fig. 2D, E). However, the HFHC+EX showed significantly attenuated NAFLD severity (mean 3.28 ± 0.95 vs. 5.00 ± 0.29 mg/mg of HFHC+EX vs. HFHC, respectively, *p* < 0.001). Additionally, the HFHC+EX demonstrated lower intrahepatic TG contents (mean 13.28 ± 2.69 vs. 18.38 ± 1.73 mg/mg of HFHC+EX vs. HFHC, respectively, *p* < 0.001), ALT (mean 131.6 ± 40.6 vs. 218.3 ± 24.9 U/L of HFHC+EX vs. HFHC, respectively, *p* < 0.001), and AST (mean 179.0 ± 29.7 vs. 229.8 ± 35.2 U/L of HFHC+EX vs. HFHC, respectively, *p* = 0.019) levels relative to the HFHC (Fig. 2). These results demonstrate that exercise training ameliorated hepatic steatosis and its impact on liver function in an animal model of HFHC-induced NAFLD.

### Pulmonary inflammation and fibrogenic patterns in HFHC-induced NAFLD

**Fig. 3:**
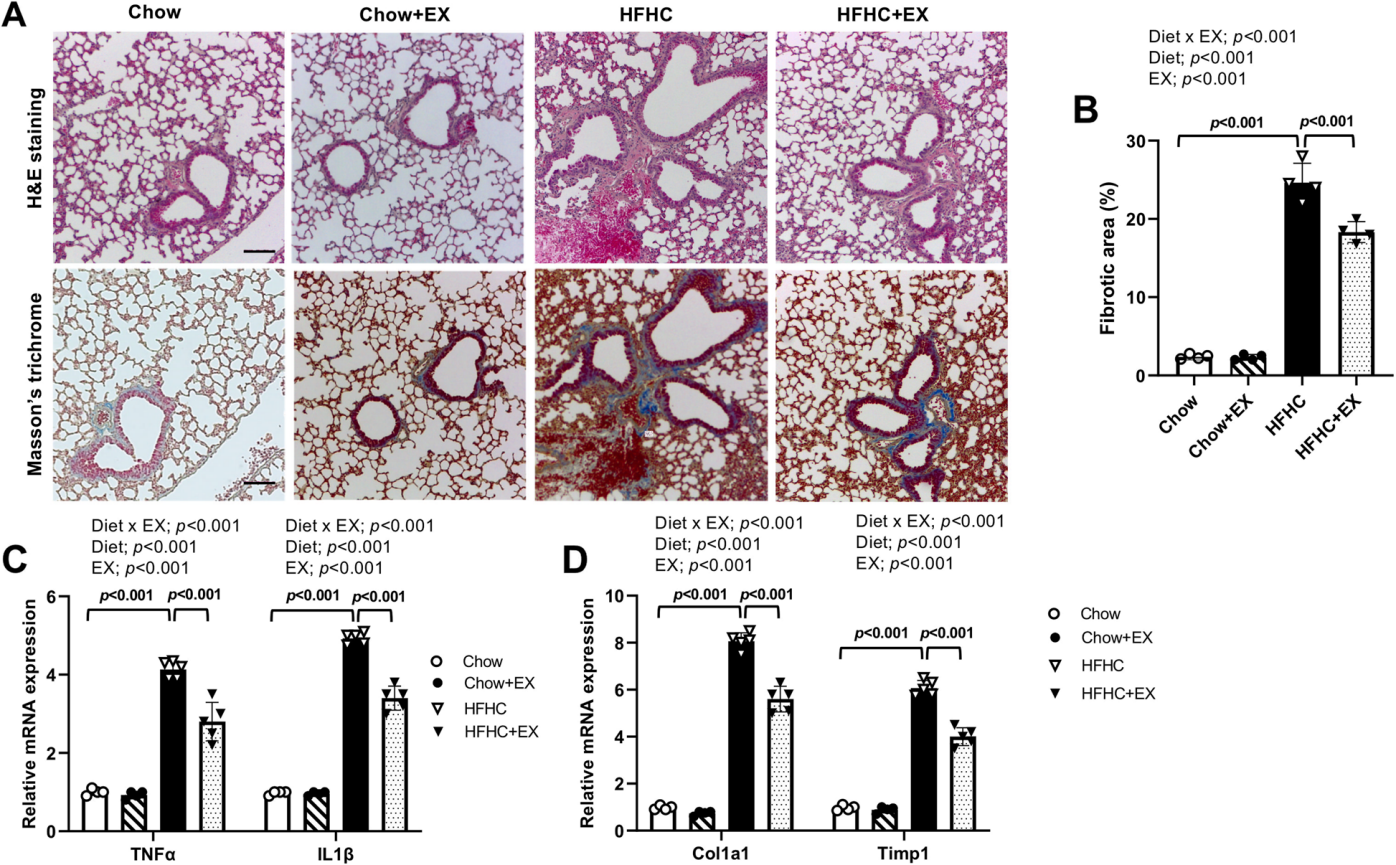
Treadmill running ameliorated the development of pulmonary inflammation and fibrogenic pattern in HFHC-induced mice. **A** Pulmonary H&E staining (upper panel) and Masson’s trichrome staining (lower panel) (*n* = 4 per group). **B** Percentage of fibrotic area (*n* = 4 per group). **C** Representative mRNA levels of genes involved in inflammation (TNF-α, IL-1β) in lung tissue (*n* = 3–5 per group). **D** Representative mRNA levels of genes involved in fibrosis (Col1a1, Timp1) (*n* = 3–5 per group). Gene expression were quantified in triplicate using real-time PCR and normalized to β-actin. Data are presented as mean ± S.D. Statistical analyses were performed by two-way ANOVA. Post hoc *t*-tests were performed to compare the differences between the 2 groups, if needed

Histological changes in lung tissue were examined by H&E and Masson’s trichrome staining. As shown in Fig. 3A, B, the HFHC-induced fibrotic deposition was increased in the lungs of HFHC-fed mice (Fig. 3B, *p* < 0.001). To confirm the histological features of collagen accumulation, the expression of inflammatory markers, including TNF-α and IL-1β, and fibrosis markers, including Col1a1 and Timp1, were investigated. There were significant differences in TNF-α, IL-1β, Col1a1, and Timp1 among the groups (*p* < 0.001). The HFHC group demonstrated significantly increased relative mRNA expression levels of TNF-α (*p* < 0.001), IL-1β (*p* < 0.001), Col1a1 (*p* < 0.001), and Timp1 (*p* < 0.001) in lung tissue compared to the Chow group (Fig. 3C, D). However, exercise training significantly attenuated HFHC-induced pulmonary inflammation and fibrosis along with alleviating the expression levels of the HFHC-related changes (Fig. 3B–D, *p* < 0.001). This suggests that exercise training had protective effects on the pulmonary inflammation and fibrosis seen in HFHC-induced NAFLD.

### Pulmonary mitochondrial function in the HFHC-induced NAFLD

**Fig. 4:**
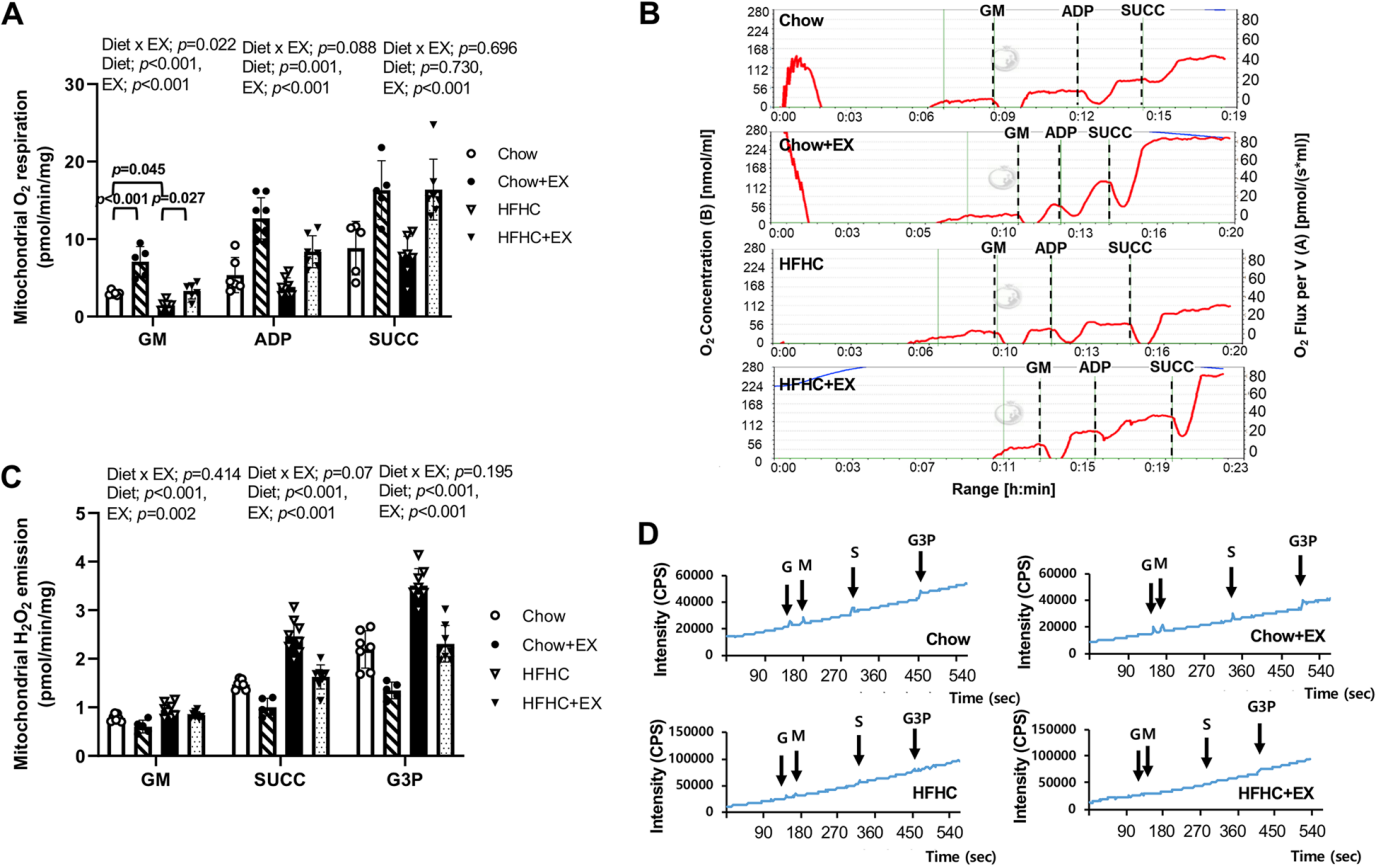
Treadmill running enhanced the pulmonary mitochondrial dysfunction in HFHC-induced mice. **A** Mitochondrial O_2_ respiration and **B** H_2_O_2_ emission normalized by wet lung tissue. *n* = 5–7 per group. Data are presented as mean ± S.D. Statistical analyses were performed by two-way ANOVA. Post hoc *t*-tests were performed to compare the differences between the 2 groups, if needed

Mitochondrial dysfunction is strongly linked to pulmonary pathophysiology [33]. To gain insight into how exercise training might affect mitochondrial function in HFHC-induced pulmonary abnormalities, mitochondrial function including mitochondrial O_2_ respiration and mitochondrial H_2_O_2_ emission in lung tissues was analyzed (Fig. 4). The mitochondrial O_2_ respiration was calculated using complex-1 (glutamate + malate, GM, state 2), ADP (state 3), and SUCC (complex-2, state 3). An HFHC diet appeared to reduce mitochondrial O_2_ respiration at the GM and ADP stages (*p* < 0.001); however, the exercise groups (Chow+EX, HFHC+EX) showed a significantly greater level of mitochondrial O_2_ respiration than the sedentary groups (Chow, HFHC) at the GM, ADP, and SUCC stages (*p* < 0.001). In addition, the level of mitochondrial O_2_ respiration appeared significantly different at the GM and a trend of significant difference at the ADP stages (*p* = 0.022 and *p* = 0.088, respectively). The HFHC had decreased mitochondrial O_2_ respiration compared to the Chow at the GM stage (*p* = 0.045) while the HFHC+EX had significantly greater mitochondrial O_2_ respiration than the HFHC at the GM stage (*p* = 0.027). However, the level of mitochondrial O_2_ respiration was not affected significantly by an HFHC diet at the SUCC stage (*p* = 0.730) and was not different among groups (*p* = 0.696).

The level of mitochondrial H_2_O_2_ emission was evaluated by basal complex-1 substrate (glutamate + malate, GM), complex-2 substrate (GM + succinate, SUCC), and lipid substrate (SUCC + glycerol-3-phosphate, G3P) (Fig. 4C, D). Although the level of the mitochondrial H_2_O_2_ emission was not different among the groups at the GM and G3P stages (*p* = 0.414 and *p* = 0.195, respectively), there was a trend of significant changes among the groups at the SUCC stage (*p* = 0.07). Furthermore, the exercise groups (Chow+EX, HFHC+EX) showed decreased mitochondrial H_2_O_2_ emission relative to the sedentary groups (Chow, HFHC) at the SUCC stage (*p* < 0.001) suggesting that exercise training attenuated oxidative stress (Fig. 4C, D).

### Pulmonary mitochondrial dynamics in the HFHC-induced NAFLD

**Fig. 5:**
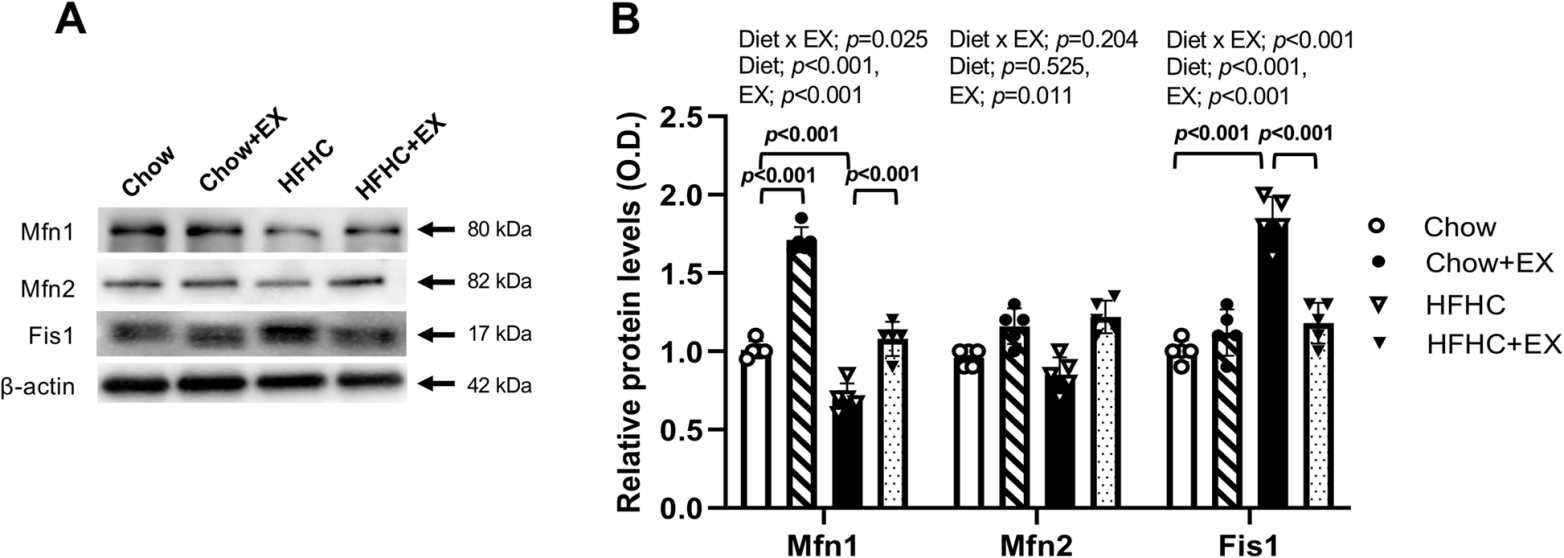
Effects of treadmill running on pulmonary mitochondrial dynamics in HFHC-induced mice. **A** Representative western blot and **B** densitometric quantitation of Mfn1, Mfn2, Fis1, and β-actin protein in the lung tissue. *n* = 4–5 per group. Data are presented as mean ± S.D. Statistical analyses were performed by two-way ANOVA. Post hoc *t*-tests were performed to compare the differences between the 2 groups, if needed. GM, glutamate+malate; G3P, glycerol-3-phosphate

The maintenance of mitochondrial homeostasis is regulated by a balance of fusion and fission of these dynamic organelles [34]. Afterward, mitochondrial dynamics were assessed by the level of fusion proteins (Mfn1, Mfn2) and fission protein (Fis 1) in the lung tissue. As shown in Fig. 5, although Mfn2 showed a non-significant difference among the groups (*p* = 0.204), Mfn 1 level showed a significant difference among the groups (*p* = 0.025). The HFHC exhibited a significant decrease in Mfn1 level relative to the Chow; however, the HFHC+EX showed a significant increase in Mfn1 compared to the HFHC (*p* < 0.001). In addition, the HFHC had significantly elevated the levels of Fis1, a mitochondrial fission protein, which were reversed by exercise training in the HFHC+EX (*p* < 0.001, Fig. 5). These data suggest that exercise training seems to be effective in restoring mitochondrial homeostasis in the lungs that can be altered by HFHC induced NAFLD.

## Discussion

The present study investigated pulmonary histological inflammation and mitochondrial dysfunction in C57BL/6 mice with HFHC-induced NAFLD and examined the impact of exercise training on these pathologic variables and associated changes in the lungs. After 15 weeks of an HFHC diet, the HFHC diet groups demonstrated obesity, hyperglycemia and impaired whole-body insulin sensitivity, elevated liver enzyme levels, and hepatic steatosis. In addition, they showed histological features of collagen accumulation in the lung tissue, increased inflammation, and reduced mitochondrial function (O_2_ respiration, H_2_O_2_ emission) with alteration of mitochondrial dynamics (Mfn1, Fis1). However, 7 weeks of exercise training was able to attenuate hepatic steatosis, reduce pulmonary inflammation, and improve mitochondrial function and dynamics in mice with HFHC-induced NAFLD.

Tumor necrosis factor-α and IL-1β are the most common inflammatory cytokines in inflammatory lung disease. In the lungs, TNF-α can arise from different cells including endothelium, macrophages, and smooth muscle cells [35]. Although the underlying mechanism is still unclear, numerous studies have reported that TNF-α is highly linked to different types of inflammatory lung disease including pulmonary fibrosis [36], asthma [37], and chronic obstructive pulmonary disease (COPD) [38]. As such, TNF-α has a significant “mediator” role in progressive lung inflammation. Moreover, TNF-α production may be further increased with NAFLD, and a study by Viglino et al. revealed that COPD with NAFLD showed a higher level of TNF-α relative to COPD without liver disease [39]. The present study revealed that 15 weeks of HFHC diet resulted in significant increases in expression levels of TNF-α mRNA. These results are in accordance with the previous findings that 10 weeks of a high-fat diet increased the frequency of asthma exacerbation along with TNF-α induced inflammatory signaling in lung tissue [40]. Furthermore, the present study demonstrated that an HFHC diet increased the expression levels of IL-1β mRNA in the lung tissue. Activation of IL-1β expression is known to induce lung inflammation by increasing infiltration of neutrophils and macrophages [40], while IL-1β blockade could ameliorate lung inflammation in high-fat diet-induced obese mice [41]. In addition to increases in inflammatory cytokines, pulmonary fibrosis was also observed in the HFHC diet groups in the present study. Moreover, compared to other groups, the HFHC showed upregulation of fibrosis-associated gene expressions such as Timp 1 (involved in extracellular matrix remodeling) and Col1a1 (involved in extracellular matrix production), which contributes to fibrogenic progression via regulation of matrix synthesis in the liver and lung tissue. Since TNF-α and IL-1β are significant mediators of Timp 1 and Col1a1 [42–44], it is speculated that these cytokine increases contributed largely to the upregulation in Timp 1 and Col1a1.

In the present study, 7 weeks of aerobic exercise training was effective at reducing the inflammatory response and fibrogenic patterns in the lung tissue by decreasing cytokine levels including TNF-α and IL-1β and fibrosis-associated gene expressions including Timp 1 and Col1a1. Previous studies have found that exercise training reduced circulating and muscular TNF-α and IL-1β levels [45, 46]. Although the exact mechanism of this beneficial effect of exercise training in the intracellular matrix is not clear, it is speculated that exercise-induced attenuation of the nuclear factor kappa B (NF-kB) signaling pathway may play a role in the downregulation of TNF-α and IL-1β. Nuclear factor kappa B is a central mediator of the inflammatory process, and both TNF-α and IL-1β gene promoter regions contain a consensus sequence for NF-kB p65 binding site [47, 48]. Previous studies reported that exercise inhibited the NF-kB signaling pathway [49], and in turn, inhibition of NF-kB could reduce inflammatory gene expressions [50]. In addition, other studies have shown that exercise attenuates the activation of NF-kB and the production of inflammatory gene expression in healthy mice and mice with lung inflammation [51, 52]. Accordingly, downregulated cytokines after 7 weeks of exercise in the present study were likely due to ameliorated NF-kB.

Nevertheless, the anti-inflammatory effect of exercise on lung tissue is still not clear. There are few similar studies reporting the effect of long-term exercise training on pulmonary inflammation. A study by Du et al. revealed that exercise training attenuated IL-1β in the lung tissues of obese mice [53], and a study by de Paula Vieira et al. found that exercise training reduced TNF-α levels in bronchoalveolar lavage fluid in mice that were exposed to air pollution [54]. Likewise, exercise-induced reductions in inflammatory cytokines contributed to attenuations in TIMP-1 and Col1a1 levels in the lung tissues in the present study. This finding, although different pathology, is concordant with previous reports that exercise training reduced TIMP-1 expression and attenuated myocardial fibrosis in rats with myocardial infarction [55]. Taken together, these results suggest that exercise training can attenuate the inflammatory reaction in the lung tissues to HFHC-induced NAFLD.

It has been suggested that mitochondrial dysfunction not only causes metabolic reprogramming of glucose metabolism but also increased profibrotic responses [33]. In the present study, an HFHC diet resulted in reduced mitochondrial O_2_ respiration in the lung tissue. Moreover, it increased fission protein (Fis1) and reduced fusion protein (Mfn1) levels. The mitochondria are dynamic organelles whose homeostasis is regulated by the balance of fusion and fission [34]. Balanced mitochondrial fusion/fission dynamics is critical for facilitating OXPHOS and optimal cellular metabolic functions, wherein fusion can rescue damaged mitochondria by blending the partially mitochondrial contents as a complementary form while fission helps enabling the elimination of damaged mitochondria and activates the mitophagy process as well as promotes mitochondrial biogenesis. However, imbalanced fusion/fission can result in the accumulation of damaged mitochondria thereby reducing OXPHOS and increasing ROS production with oxidized mitochondria DNA (mtDNA) build-up. mtDNA per se acts as an inflammation mediator that interacts with circulating neutrophils resulting in alteration of the transcriptional rate of pro-inflammatory cytokines such as TNF-α and IL-1β through activated NF-kB [56–58]. Indeed, the present study observed increased mitochondrial H_2_O_2_ emission in lung tissue in the HFHC diet groups. Elevated H_2_O_2_ production due to mitochondrial dysfunction consequently develops pulmonary fibrosis [59]. This is consistent with the increased fibrogenic pattern observed in the present study. In addition, it is supported by a previous study reporting that high-fat diet impaired mitochondrial function and increased fibrogenic response [60]. High-fat availability and NAFLD-induced oxidative stress may be essential components of inflammatory respiratory pathology [9]. As a result, reductions in mitochondrial function and dynamics will alter mitochondrial homeostasis, increase ROS production, and facilitate the fibrogenic progression. Chronically, increased ROS exposure will cause further mitochondrial dysfunction, more severe apoptosis and lung cell damage [61], and consequently fibrosis progression [62].

In the present study, exercise training alleviated deteriorations in mitochondrial function and dynamics in lung tissue resulting from an HFHC diet to induce NAFLD. Relative to the sedentary groups, the exercise groups demonstrated improved mitochondrial O_2_ respiration and H_2_O_2_ emission. In addition, the HFHC+EX increased fusion proteins (Mfn1) and reduced fission protein (Fis1) than the HFHC. Parallel results were observed by other studies, which investigated the expression of Mfn1 and Mfn2 in skeletal muscle tissues in animal [63] and human models [64]. The underlying mechanism of the effects of exercise training on mitochondria in the lung cells is complicated. Previous studies demonstrated that aerobic exercise training resulted in reduced H_2_O_2_ production, reduced ROS production, and increased antioxidant capacity in skeletal muscle cells including the diaphragm [65–67]. In addition, exercise training seems to improve mitochondrial function by using electrons more efficiently at the respiratory chain level rather than improving the ability to weaken ROS generation [68]. Another study has also reported that exercise training improved mitochondrial biogenesis in lung tissue followed by a reduction in mitochondrial ROS production, consistent with our findings [69].

The present study has limitations. Although the present study determined the beneficial effects of exercise training on mitochondrial function and mitochondrial dynamics in lung tissues with HFHC-induced NAFLD, mitochondrial morphology in lung tissue was not assessed. However, given that decreased fusion protein and increased fission protein are more likely to contribute to the proliferation/apoptosis imbalance in lung diseases such as COPD, asthma, and cancer [70], it is speculated that mitochondrial dynamics can be largely explained by the quantification of mitochondrial dynamic protein. In addition, although the present study demonstrates the effect of exercise training on pulmonary pathophysiology in HFHC-induced NAFLD model mice, it did not elucidate the direct mechanistic relationship between the lung and the liver.

## Conclusions

In the present study, an HFHC diet caused greater pulmonary inflammation and fibrosis and reduced mitochondrial function and dynamics compared to the other experimental groups. These pathologic alterations in pulmonary vascular and parenchymal tissues may significantly contribute to the decline in pulmonary function and physiology often observed in patients with NAFLD. However, 7 weeks of exercise training was able to attenuate those pathophysiologic alterations. To the best of our knowledge, these findings on the effects of exercise training on pulmonary mitochondrial homeostasis in HFHC-induced NAFLD are novel. The results of this study suggest that exercise may be a nonpharmacological option or a synergistic option with pharmacological treatment for pulmonary dysfunction in NAFLD.

## Supplementary Information

**Additional file 1.** The ARRIVE Essential 10: Compliance Questionnaire.

**Additional file 2.**

**Additional file 3.**

## Data Availability

The datasets generated and/or analyzed during the current study are not publicly available but are available from the corresponding author upon reasonable request.

## Abbreviations

ADP: Adenosine diphosphate
ALT: Alanine aminotransferase
AST: Aspartate transaminase
AUCs: Area under the curve
Chow: Standard diet
Chow+EX: Standard diet with exercise
Col1a1: Collagen type I alpha 1 chain
COPD: Chronic obstructive pulmonary disease
Fis 1: Fission protein
GTT: Glucose tolerance test
H_2_O_2_: Hydrogen peroxide
HFHC: High-fat high-carbohydrate diet
HFHC+EX: High-fat high-carbohydrate diet with exercise
IL-1β: Interleukin-1β
Mfn 2: Mitofusin 1 (Mfn 1) and 2
NAFLD: Non-alcoholic fatty liver disease
NASH: Non-alcoholic steatohepatitis
ROS: Reactive oxygen species
SUCC: Succinate
Timp1: TIMP metallopeptidase inhibitor 1
TNF-α: Tumor necrosis factor-α
β-actin: Beta-actin
