# Supplementary figures and images for: Exercise training attenuates pulmonary inflammation and mitochondrial dysfunction in a mouse model of high-fat high-carbohydrate-induced NAFLD
Source: BMC Med. 2022 Nov 8;20:429. doi: 10.1186/s12916-022-02629-1 (PMC9644617; doi:10.1186/s12916-022-02629-1)

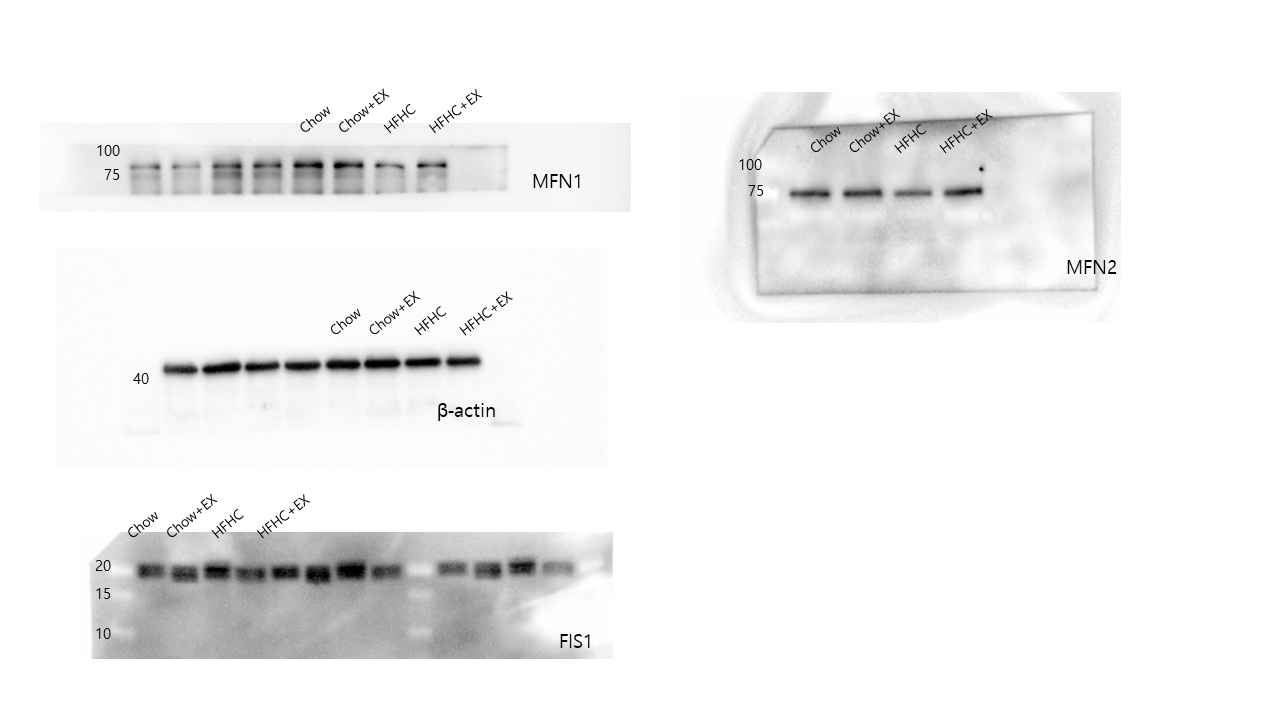

Supplement: Supplementary file 2 — Additional file 2. [file 12916_2022_2629_MOESM2_ESM.tif]

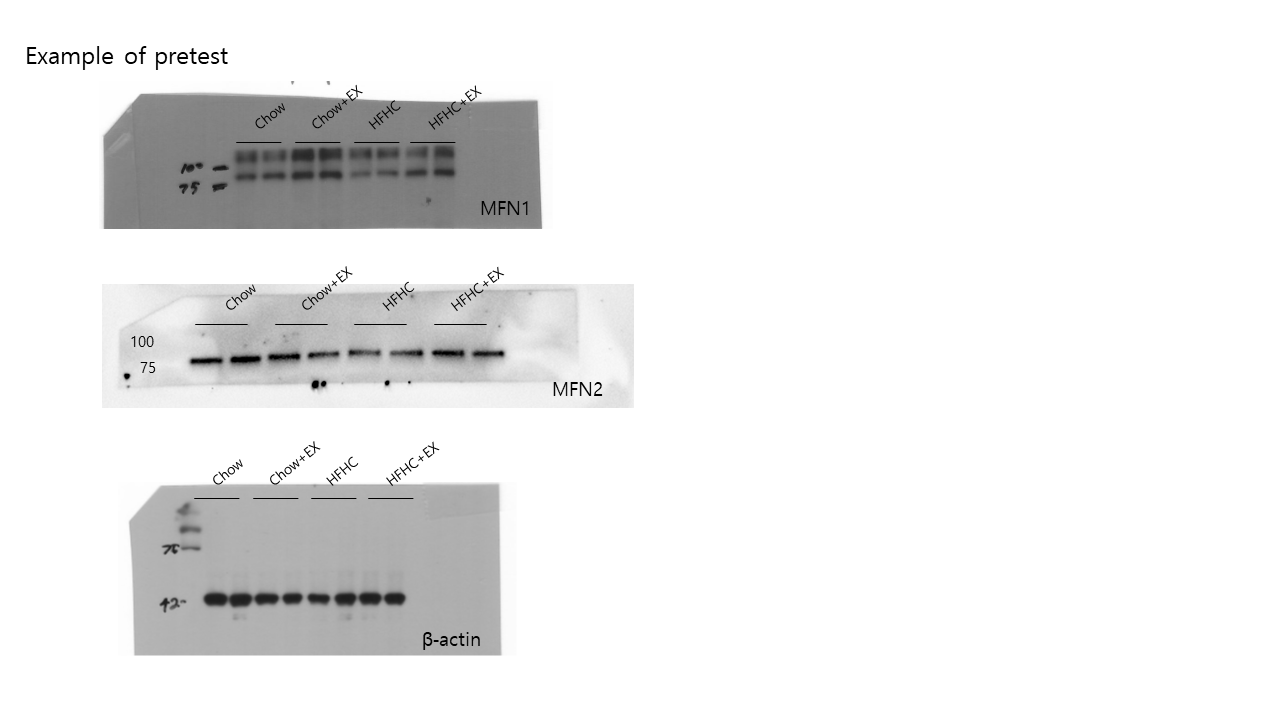

Supplement: Supplementary file 3 — Additional file 3. [file 12916_2022_2629_MOESM3_ESM.tif]
